# Management of Talons Cusp associated with Primary Central Incisor: A Rare Case Report

**DOI:** 10.5005/jp-journals-10005-1153

**Published:** 2012-08-08

**Authors:** Rakesh N Bahadure, Nilima Thosar, Eesha S Jain

**Affiliations:** Lecturer, Department of Pedodontics and Preventive Dentistry Sharad Pawar Dental College, Wardha, Maharashtra, India; Professor and Head, Department of Pedodontics and Preventive Dentistry, Sharad Pawar Dental College, Wardha, Maharashtra, India e-mail: drnthosar@rediffmail.com; Postgraduate Student, Department of Pedodontics and Preventive Dentistry, Faculty of Dental Sciences, Chhatrapati Shahuji Maharaj Medical University, Lucknow, Uttar Pradesh, India

**Keywords:** Talons cusp, Primary maxillary left central incisor, Pulpectomy

## Abstract

The talon cusp is a relatively rare dental developmental anomaly characterized by cusp-like projections, usually observed on the lingual surface of the affected tooth. Normal enamel covers the cusp and fuses with the lingual aspect of the tooth. The cusp may or may not contain an extension of the pulp. The condition can occur in both the primary and permanent dentitions. However, the occurrence of anomalous cusp is rather infrequent in the primary dentition. Little data is available about the treatment of talon cusps in the primary dentition as compared with the permanent dentition. A case of talon cusp in the primary maxillary left central incisor is reported. This dental anomaly was not associated with any other somatic or dental abnormality. The tooth was carious. Pulpal extension into the cusp was detected radiographically. Pulpectomy of the tooth was carried out and restored with composite restoration.

**How to cite this article:** Bahadure RN, Thosar N, Jain ES. Management of Talons Cusp associated with Primary Central Incisor: A Rare Case Report. Int J Clin Pediatr Dent 2012;5(2): 142-144.

## INTRODUCTION

Talon cusp is defined as an accessory cusp-like structure projecting from the lingual surface of a primary or permanent anterior tooth and extending at least half the distance from the cementoenamel junction to the incisal edge.^1^ A review of literature suggests that the talon cusp has a striking predilection for the maxilla over the mandible with the majority of the cases occurring in maxillary anterior teeth.^2^ It may present unilaterally or bilaterally in males or females.^3^ Talon cusp occurs more frequently in permanent than primary dentitions^4^ and shows a predilection for the maxilla over the mandible.^4^ The maxillary lateral incisors are the most commonly affected (67%) followed by the central incisors (24%) and canines (9%).^5^ In the majority of cases, the talon cusp is originated from the lingual surface of the tooth.^6^

The etiology of the talon cusp remains unknown. It has been suggested that it may have a multifactorial etiology to include genetic, environmental factors and hyperactivity of the dental lamina early in odontogenesis.^7^

The present case report describes the clinical management of carious talon cusp along with pulp exposure on the primary maxillary left central incisor.

## CASE REPORT

**Fig. 1:**
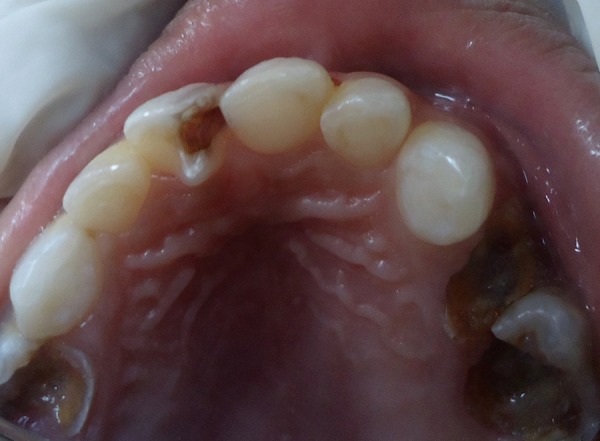
Talon cusp

**Fig. 2:**
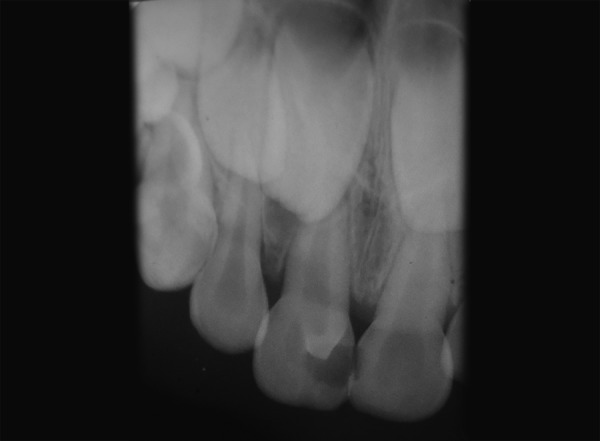
Radiographic view of talon cusp

**Fig. 3:**
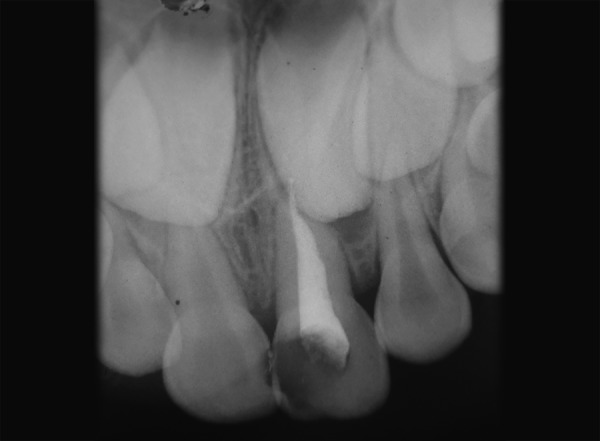
Treated case of talon cusp with pulpectomy

**Fig. 4:**
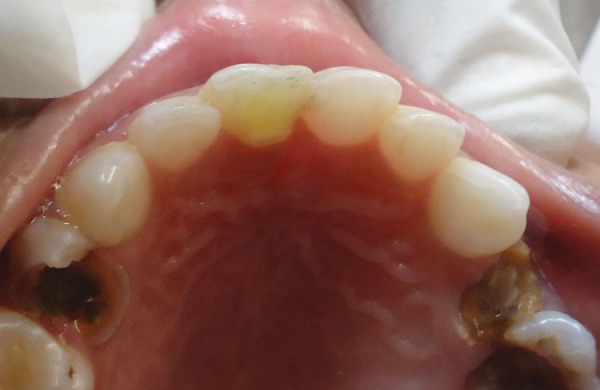
Grinded and restored cusp in normal form

A 4-year-old male child reported to the Department of Pedodontics and Preventive Dentistry with the chief complaint of pain in upper left front teeth region. The patient's medical history was noncontributory. Examination of the oral cavity revealed normal soft tissue and normal development of primary dentition. An anomalous cusp-like structure was detected on the palatal surface of primary maxillary right central incisor that extended from the cervical margin of the tooth toward the incisal edge with more inclination toward mesial surface forming triangular spike like projection involved with caries (Fig. 1). An intraoral periapical radiograph of this tooth revealed the additional cusp with its pulpal extension (Fig. 2). There was no history of intraoral swelling associated with tooth. Deep carious lesion observed on mesial and palatal surfaces. Depending upon the signs and symptoms of the patient, pulpectomy of 61 was considered necessary. A pulpectomy was performed. The canal was filled with metapex (Meta Biomed, Korea) and the talon cusp was then removed and the tooth was restored with composite resin restoration to its common morphology (Figs 3 and 4). The composite resin restoration was polished and checked for occlusal interferences. Upon follow-up examination 1 month after the procedure, no adverse signs or symptoms and no periradicular pathology were noted. Patient was scheduled for regular check-up examinations. Subsequent clinical and radiographic recall examinations confirmed no signs of pathosis. A periapical radiograph showed normal root resorption of both primary central incisors.

International Journal of Clinical Pediatric Dentistry, May-August 2012;5(2):142-144

## DISCUSSION

The first case was reported by Mitchell in 1892 on the lingual surface of a maxillary central incisor, who described it as ‘a process of horn like shape curving from the base downward to the cutting edge'.^8^ The term talon cusp was coined because of its resemblance to an eagle's talon in shape.^9^

Although talon cusp usually occurs as an isolated entity, its incidence has reportedly increased in teeth related to cleft palate syndromes and in association with other anomalies.^10^ The case reported here was not associated with any known abnormal systemic developmental syndrome.

A more detailed classification of talon cusps was proposed by Hattab et al ^5^ who classified talon cusps into three types. A type 1 (major) talon was defined as a morphologically well-defined additional cusp that projects from the facial and/or palatal/lingual surface of an anterior tooth and extends at least half the distance from the cementoenamel junction to the incisal edge. A type 2 (minor) talon extends more than one-fourth, but less than half the distance from the cementoenamel junction to the incisal edge, while a type 3 (trace) talon is an enlarged or prominent cingulum and its variations which occupy less than one-fourth the distance from the cementoenamel junction to the incisal edge. In the present case, talon cusp extended from cingulum including more than half of the tooth structure up to the level of incisal edge.

The extent of pulp extension into the cusp is however, difficult to determine because of its superimposition over the main pulp chamber.^11^ While some indicated that talon cusps contain pulp tissue,^9^ some found no evidence of pulp extension into the cusp.^12^ However, it has been suggested that large talon cusps, especially those that stand away from the tooth crown are more likely to contain pulp tissue.^13,11^

The presence of a talon cusp is not always an indication for dental treatment unless it is associated with problems, such as compromised esthetics, occlusal interference, tooth displacement, caries, periodontal problems or irritation of the soft tissues during speech or mastication.^14,6^ Severe attrition or fracture of the enamel surface can cause exposure of the dentine-pulp complex and consequently, pulp necrosis.^15^ In this case, the cusp was prominent and sharply defined and projected from the cervical region to the incisal edge of the tooth. Pulpal involvement of talon cusp could be because of the deep groove which joined the cusp to the tooth acted as stagnation areas for plaque and debris to become carious. So due to pulpal involvement of carious talon cusp, patient experienced pain. Pulpectomy of 61 with talon cusp was done. Therefore, early recognition and diagnosis is important so that intervention can be done at initial stage itself.
